# Antigen-specific human NKT cells from tuberculosis patients produce IL-21 to help B cells for the production of immunoglobulins

**DOI:** 10.18632/oncotarget.5764

**Published:** 2015-09-21

**Authors:** Changyou Wu, Zitao Li, Xiaoying Fu, Sifei Yu, Suihua Lao, Binyan Yang

**Affiliations:** ^1^ Institute of Immunology, Zhongshan School of Medicine, Guangdong Provincial Key Laboratory of Organ Donation and Transplant Immunology, Sun Yat-Sen University, Guangzhou, China; ^2^ Chest Hospital of Guangzhou, Guangzhou, China

**Keywords:** NKT cells, tuberculosis, IL-21, B cells, immunoglobulins, Immunology and Microbiology Section, Immune response, Immunity

## Abstract

Natural killer T (NKT) cells from mouse and human play an important role in the immune responses against *Mycobacterium tuberculosis*. However, the function of CD3^+^TCRvβ11^+^ NKT cells at the local site of *M. tuberculosis* infection remains poorly defined. In the present study, we found that after stimulation with *M. tuberculosis* antigens, NKT cells isolated from tuberculosis (TB) pleural fluid mononuclear cells (PFMCs) produced IL-21 and other cytokines including IFN-γ, TNF-α, IL-2 and IL-17. IL-21-expressing NKT cells in PFMCs displayed effector memory phenotype, expressing CD45RO^high^CD62L^low^CCR7^low^. Moreover, NKT cells expressed high levels of CXCR5 and all of IL-21-expressing NKT cells co-expressed CXCR5. The frequency of BCL-6-expression was higher in IL-21-expressing but not in non-IL-21-expressing CD3^+^TCRvβ11^+^ NKT cells. Sorted CD3^+^TCRvβ11^+^ NKT cells from PFMCs produced IFN-γ and IL-21 after stimulation, which expressed CD40L. Importantly, CD3^+^TCRvβ11^+^ NKT cells provided help to B cells for the production of IgG and IgA. Taken together, our data demonstrate that CD3^+^TCRvβ11^+^ NKT cells from a local site of *M. tuberculosis* infection produce IL-21, express CXCR5 and CD40L, help B cells to secrete IgG and IgA, and may participate in local immune responses against *M. tuberculosis* infection.

## INTRODUCTION

Tuberculosis (TB), one of the oldest infectious diseases associated with humans, is a chronic disease caused by infection with *Mycobacterium tuberculosis* [1, 2]. The incidence of TB has increased during the past 20 years for reasons such as insufficient prevention efforts, incorrectly prescribed medication, the emergence of drug-resistant strains of *M. tuberculosis* and the prevalence of human immunodeficiency virus (HIV) infection [3, 4]. In 2011, there were an estimated 8.7 million new cases of TB, and the disease was responsible for roughly 1.4 million deaths [5].

Human natural killer T (NKT) cells are a rare subset of T lymphocytes and are characterized by their restricted expression of an invariant Vα24-Jα18 T cell receptor (TCR) chain paired with the Vβ11 TCR chain. This pair of TCR chains recognizes glycolipid antigens, such as α-galactosylceramide (α-GalCer), presented by the major histocompatibility complex (MHC) class I-like molecule CD1d [6]. NKT cells can rapidly produce very large amounts of cytokines, including interferon-γ (IFNγ), interleukin-4 (IL-4), IL-10, IL-13, IL-17, IL-21 and tumour necrosis factor (TNF) following stimulation, and they are able to either promote or suppress cell-mediated immunity without the need for clonal expansion [7, 8]. Quantitative and qualitative defects in the NKT cell pool, NKT cells inappropriately reactive with self (or non-self) glycolipid antigens, and NKT-derived cytokines have been associated with occurrence of diseases.

IL-21 is predominantly produced by activated CD4^+^ T cells and natural killer (NK) T cells [9-11]. IL-21 exerts many biological actions. IL-21 can induce the activation, proliferation and differentiation of T cells, NK cells and NKT cells, and promotes proliferation and differentiation of the macrophage and granulocyte lineages [12]. IL-21 has potent anti-tumor activity by activating CD8^+^ T cells and NKT cells [13]. Several studies reported the role of IL-21 in the pathogenesis of systemic lupus erythematosus (SLE) and rheumatoid arthritis (RA) [14-17]. A report describing novel sequence variants in genes encoding IL-21 and the IL-21R indicates that polymorphisms within IL-21 and the IL-21 receptor are positively associated with type 1 diabetes in humans [18].

Emerging evidence has shown that murine and human NKT cells may mediate protection against *M. tuberculosis* [19-23]. For example, it was demonstrated in a recent study that α-GalCer administration, alone or in combination with classic chemotherapy, can improve the clinical outcomes of *M. tuberculosis* infection in mice [22]. It has also been shown that α-GalCer incorporation into bacillus Calmette-Guérin (BCG) vaccine enhances the host immune response by modulating T cell priming *via* murine NKT cell activation [23]. Although a numerical deficiency of NKT cells has been found in the patients with pulmonary TB [24-26], much less is known about the frequency of human NKT cells and their functions in patients with *M. tuberculosis* infection. It has been recently reported that NKT cells produce very high levels of IL-21 following BCG immunization in mice and humans [27]. Children with active TB, compared with healthy controls, showed markedly diminished production of type 1 (IFN-γ and TNF-α), 2 (IL-4 and IL-13), and 17 (IL-17A, IL-21, and IL-23)-associated cytokines [28, 29].

In this study, we demonstrate for the first time that NKT cells isolated from pleural fluid mononuclear cells (PFMCs) from TB patients produce IL-21 following stimulation with *M. tuberculosis* (Mtb)-specific antigens and that IL-21 is able to induce the production of IgG and IgA by B cells, which might influence the local immune response to *M. tuberculosis* in TB patients.

## RESULTS

### The frequency of IL-21-expressing CD3^+^TCRvβ11^+^ NKT cells in PFMCs and PBMCs, and relationship between IL-21, IFN-γ and IL-17 expression by CD3^+^TCRvβ11^+^ NKT cells from PFMCs

**Figure 1:**
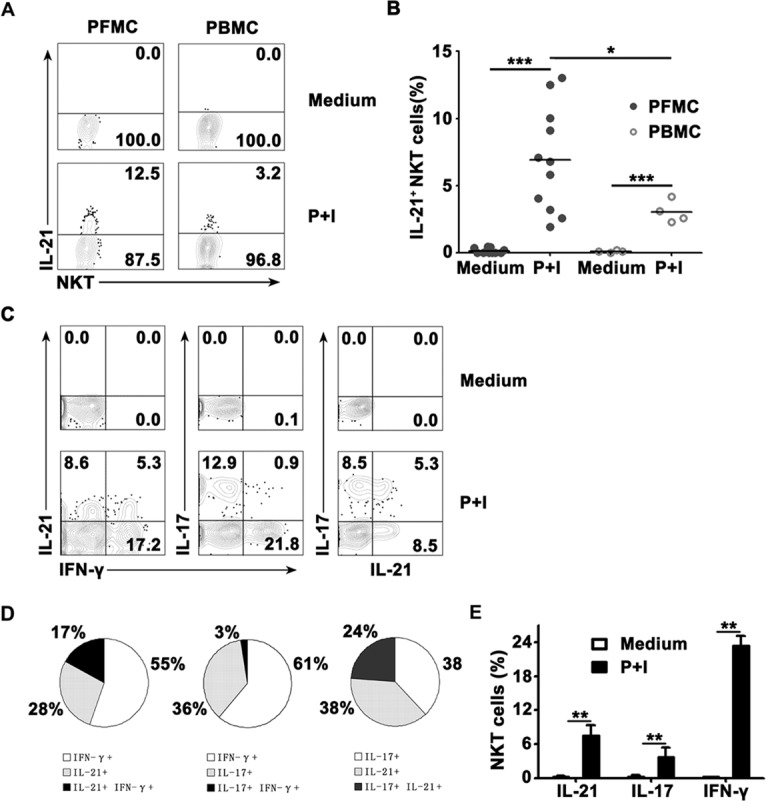
The frequency of IL-21- expressing TCRvβ11^+^ NKT cells in PFMCs and PBMCs, and the expression of IL-21 with IFN-γ and IL-17 in CD3^+^TCRvβ11^+^ NKT cells Freshly isolated PFMCs and PBMCs were stimulated for 6 hrs with or without PMA plus Ionomycin and stained with anti-CD3 and anti-TCRvβ11 for the analysis of the expression of IL-21, IFN-γ and IL-17. **A.** Expression of IL-21 by NKT cells was detected by FACS. **B.** Statistical results of the frequency of IL-21^+^ NKT cells in PFMCs and PBMCs. **C.** Representative data are shown for the relationships between IL-21, IFN-γ and IL-17 expression. **D.** The percentages of IL-21, IL-17 and IFN-γ- expressing NKT cells. **E.** Statistical results for the frequency of cytokine-producing NKT cells following stimulation with PMA plus Ionomycin, as compared to unstimulated NKT cells. *, *P*,< 0.05; **, *P <* 0.01; ***, *P <* 0.001.

To identify whether CD3^+^TCRvβ11^+^ NKT cells from PFMCs and PBMCs could produce IL-21, freshly isolated PFMCs from tuberculous pleural effusion and PBMCs from venous blood were stimulated *in vitro* with PMA plus Ionomycin. After six hours, CD3^+^TCRvβ11^+^ NKT cells from PFMCs and PBMCs were gated and analyzed for the expression of IL-21 by flow cytometry. As shown in Figure 1A, without any stimulation, CD3^+^TCRvβ11^+^ NKT cells from PFMCs and PBMCs did not express IL-21. Following stimulation with PMA plus Ionomycin, CD3^+^TCRvβ11^+^ NKT cells from PFMCs and PBMCs expressed IL-21. The mean frequency of CD3^+^TCRvβ11^+^IL-21^+^ NKT cells in PFMCs and in PBMCs was 6.8% (ranging from 1.95% to 13.01%) and 3.05% (ranging from 2.3% to 4.2%) (Figure 1B), respectively. Furthermore, the frequency of IL-21-expressing CD3^+^TCRvβ11^+^ NKT cells in PFMCs was significantly higher than in PBMCs (*P* < 0.05). To clarify the relationships between the production of IL-21, IFN-γ, and IL-17 by CD3^+^TCRvβ11^+^ NKT cells from PFMCs, freshly isolated PFMCs were cultured with PMA plus Ionomycin for 6 hrs. NKT cells were stained for further analysis of the expression of IL-21, IFN-γ and IL-17. Representative data are shown in Figure 1C to illustrate relationships between the expression of IL-21, IFN-γ and IL-17 by NKT cells. As shown in Figure 1D, after stimulation with PMA plus Ionomycin, about 7.5% of CD3^+^TCRvβ11^+^ NKT cells expressed IL-21, 3.6% of them expressed IL-17 and 23.4% of them expressed IFN-γ. Among the subset of NKT cells that expressed IL-21 and/or IFN-γ, 28% expressed IL-21 but not IFN-γ, 17% expressed both IL-21 and IFN-γ, and 55% expressed IFN-γ but not IL-21. Thirty-six percent of the cells expressed IL-17, 3% of cells expressed both IL-17 and IFN-γ, and 61% of cells expressed IFN-γ only. In addition, 38% of the cells expressed either IL-21 or IL-17, and 24% of cells expressed both IL-17 and IL-21 (Figure 1E).

### PPD induced the production of IL-21, IFN-γ, TNF-α, IL-2 and IL-17 by CD3^+^TCRvβ11^+^ NKT cells from PFMCs

**Figure 2:**
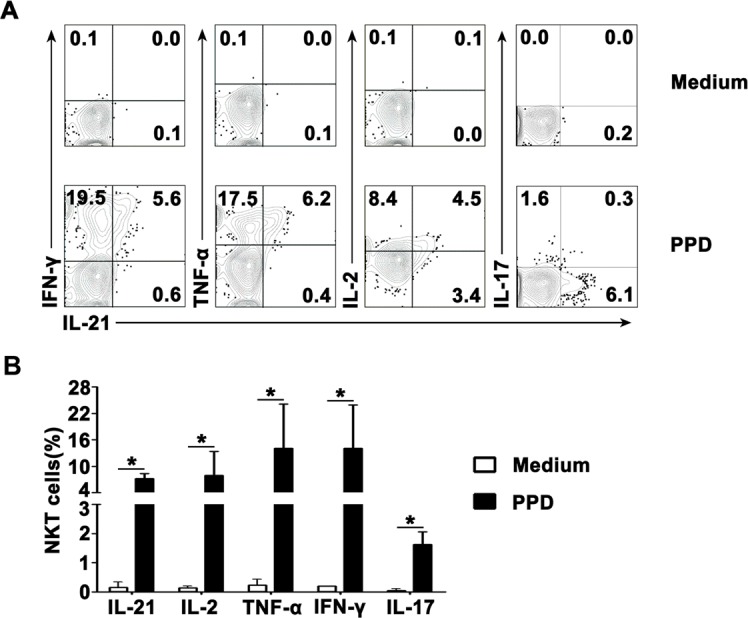
Antigen-induced expression of IL-21 with IFN-γ, TNF-α, IL-2 and IL-17 by CD3^+^TCRvβ11^+^ NKT cells PFMCs were incubated for 6 hrs with or without PPD plus anti-CD28 NKT cells were gated and analyzed for the expression of cytokines. **A.** Statistical results of the expression of IL-21, IFN-γ, TNF-α, IL-2 and IL-17 after stimulation with PPD plus anti-CD28 are shown. *, *P* < 0.05. **B.** The expression of IL-21 with IFN-γ, TNF-α, IL-2 and IL-17 are shown. Data are representative of three independent experiments.

In order to demonstrate whether PPD could induce expression of IL-21, IFN-γ, TNF-α, IL-2 and IL-17 by CD3^+^TCRvβ11^+^ NKT cells from PFMCs, freshly isolated PFMCs were cultured *in vitro* with PPD plus anti-CD28 mAb. After stimulation for 6-8 hrs. NKT cells were defined by staining with anti-CD3 and anti-TCRvβ11, and the expression of IL-21, IFN-γ, TNF-α, IL-2 and IL-17 by CD3^+^TCRvβ11^+^ NKT cells from PFMCs was analyzed by flow cytometry. The statistical results in Figure 2A showed that after stimulation with PPD plus anti-CD28, 7.2% of CD3^+^TCRvβ11^+^ NKT cells expressed IL-21, 7.9% of CD3^+^TCRvβ11^+^ NKT cells expressed IL-2, 14.1% of CD3^+^TCRvβ11^+^ NKT cells expressed TNF-α, 14.0% of CD3^+^TCRvβ11^+^ NKT cells expressed IFN-γ and 1.6% of CD3^+^TCRvβ11^+^ NKT cells expressed IL-17. In addition, the representative data shown in Figure 2B demonstrate that PPD induced co-expression of IL-21 with IFN-γ, TNF-α, IL-2 and IL-17 by CD3^+^TCRvβ11^+^ NKT cells. The percentages of CD3^+^TCRvβ11^+^ NKT cells that expressed IL-2 but not IL-21, or IL-21 but not IL-2, were 51.5% and 20.9%, respectively. The percentage of CD3^+^TCRvβ11^+^ NKT cells that co-expressed IL-21 and IL-2 was 27.6%. In regard to coexpression of IL-21 and IL-17, IL-21-expressing CD3^+^TCRvβ11^+^ NKT cells were different from IL-17-expressing CD3^+^TCRvβ11^+^ NKT cells completely. We also evaluated the relationships between expression of IL-21 and expression of each of the other cytokines mentioned above (Figure 2B).

### IL-21-expressing CD3^+^TCRvβ11^+^ NKT cells in PFMCs displayed memory phenotypes

**Figure 3:**
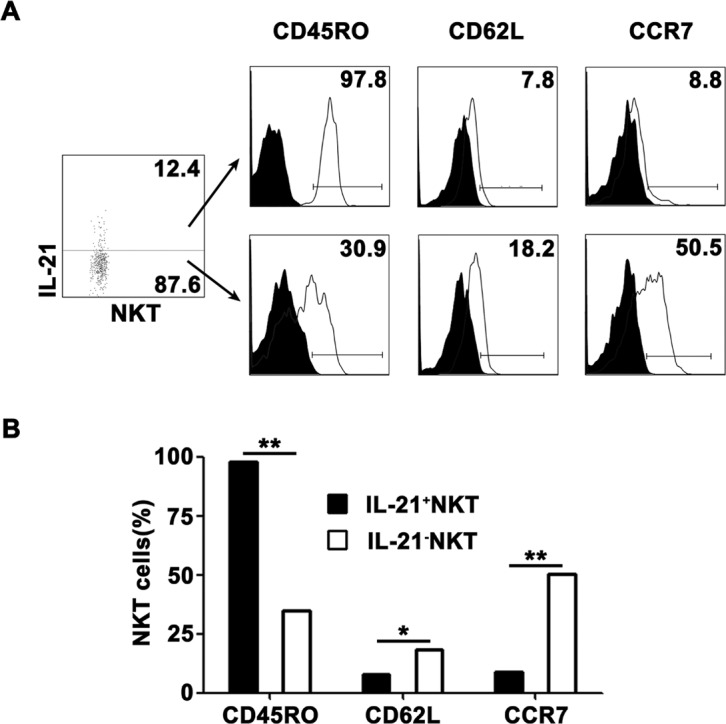
Characterization of IL-21-expressing NKT cells PFMCs were stimulated for 6 hrs with PPD plus anti-CD28, the cells were stained for NKT cells and analyzed for phenotypes of NKT cells **A.** IL-21^+^ NKT and IL-21^−^ NKT cells were gated for the analysis of the expression of CD45RO, CD62L and CCR7. **B.** Statistical results of the percentages of CD45RO, CD62L and CCR7 on IL-21^+^ NKT and IL-21^−^ NKT cells. *, *P* < 0.05; **, *P* < 0.01.

To demonstrate phenotypic characteristics of IL-21-expressing CD3^+^TCRvβ11^+^ NKT cells, freshly isolated PFMCs were stimulated with PPD plus anti-CD28, and IL-21-expressing and non-IL-21-expressing CD3^+^TCRvβ11^+^ NKT cells were gated for the analysis of the expression of CD45RO, CD62L and CCR7. The results in Figure 3A showed that nearly all of IL-21-expressing CD3^+^TCRvβ11^+^ NKT cells expressed CD45RO and thus displayed the phenotype of memory cells, and a few of them expressed CD62L and CCR7. These results indicated that IL-21-expressing CD3^+^TCRvβ11^+^ NKT cells were effector memory cells. However, many of the non-IL-21-producing CD3^+^TCRvβ11^+^ NKT cells displayed phenotypes of central memory cells. Statistical results in Figure 3B showed that IL-21-expressing CD3^+^TCRvβ11^+^ NKT cells contained a higher frequency of CD45RO-expressing cells but lower frequencies of CD62L- and CCR7-expressing cells compared to non-IL-21-producing CD3^+^TCRvβ11^+^ NKT cells.

### All of PPD-specific IL-21-expressing CD3^+^TCRvβ11^+^ NKT cells in PFMCs expressed CXCR5

**Figure 4:**
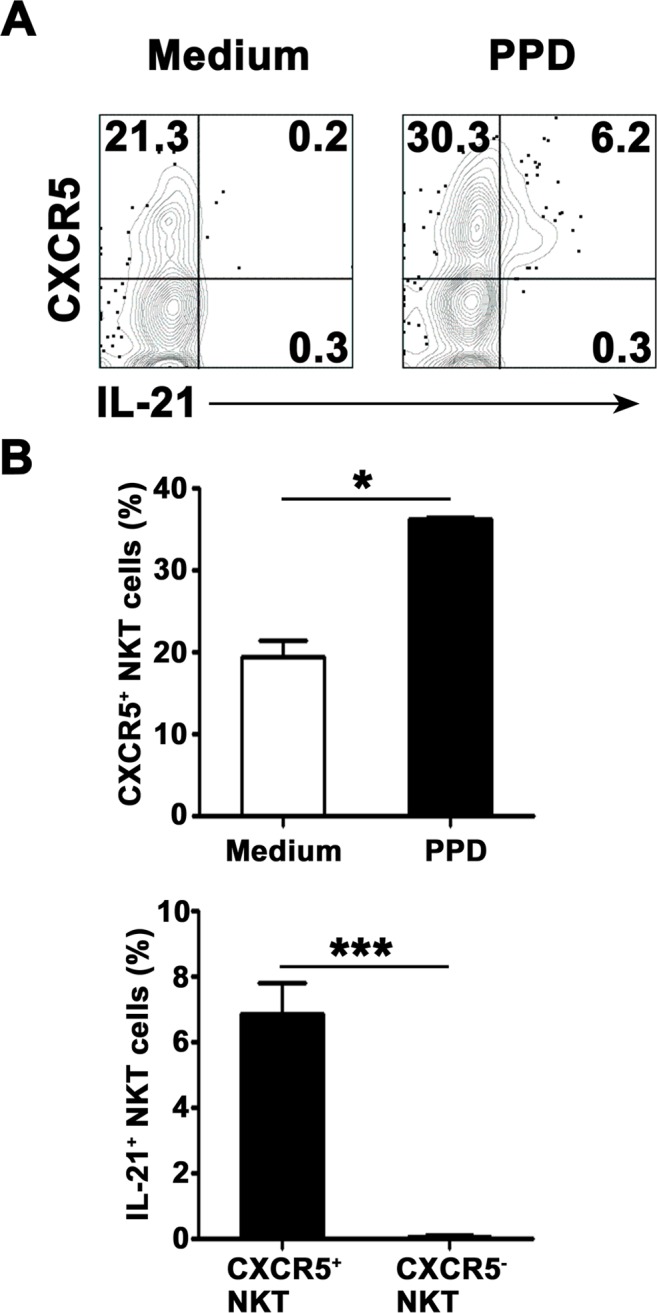
IL-21-expressing NKT cells co-expressed CXCR5 PFMCs were stimulated for 6 hrs with or without PPD plus anti-CD28 The cells were stained, gated on NKT cells and analyzed for the expression of CXCR5 on IL-21^+^ NKT cells. **A.** The expression of CXCR5 and IL-21 were measured by FACS. Data are representative of three independent experiments. **B.** Statistical results of the expression of CXCR5 on NKT cells and the frequency of IL-21-expressing cells among CXCR5^+^ and CXCR5^−^ NKT cells. *, *P* < 0.05; ***, *P* < 0.001.

To observe expression of CXCR5 on surface of CD3^+^TCRvβ11^+^ NKT cells before and after culture with PPD plus anti-CD28, freshly isolated PFMCs were stained with anti-CXCR5 mAb before stimulation or together with anti-IL-21 mAb after stimulation. The results in Figure 4A showed that after stimulation with PPD plus anti-CD28, CD3^+^TCRvβ11^+^ NKT cells expressed IL-21, and IL-21-expressing CD3^+^TCRvβ11^+^ NKT cells co-expressed CXCR5. Statistical results in Figure 4B showed that after stimulation, the percentage of CD3^+^TCRvβ11^+^ NKT cells expressing CXCR5 was increased. All of IL-21-expressing CD3^+^TCRvβ11^+^ NKT cells expressed CXCR5.

### Nearly all of PPD-specific IL-21-expressing CD3+TCRvβ11+ NKT cells in PFMCs coexpressed CD40L

**Figure 5:**
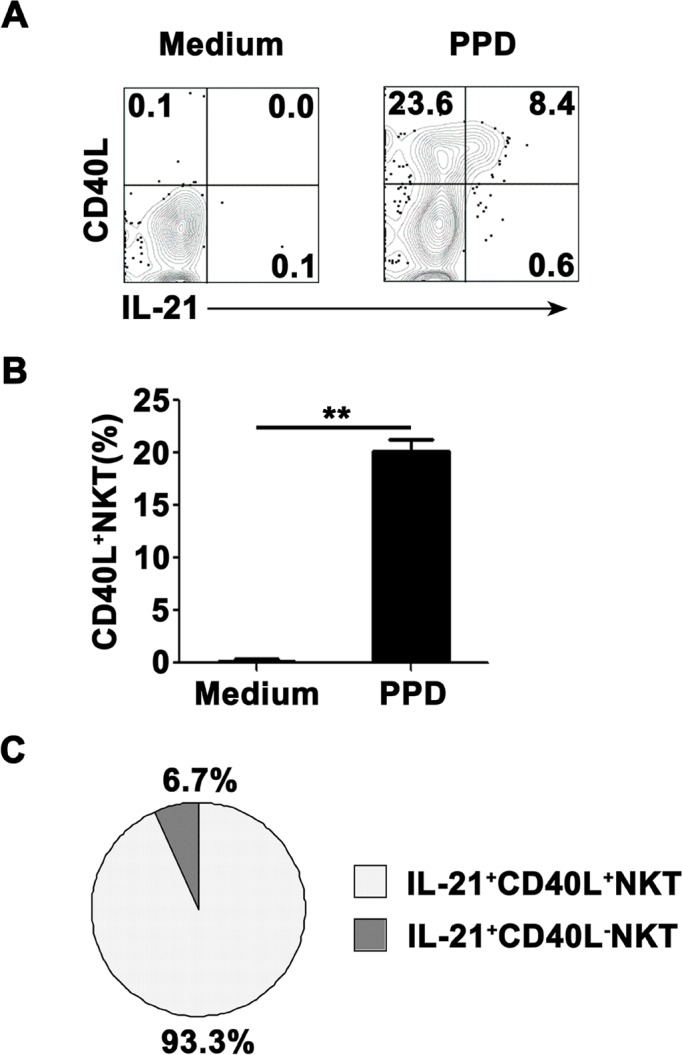
The expression of CD40L on IL-21- expressing NKT cells PFMCs were stimulated for 6 hrs with or without PPD plus anti-CD28 The cells were stained, gated on NKT cells and analyzed for the expression of CD40L on IL-21^+^ NKT cells. **A.** The expression of CD40L and production of IL-21 were detected by FACS. Data are representative of three independent experiments. **B.** Statistical results of the expression of CD40L on NKT cells. **, *P* < 0.01. **C.** Results of the expression of CD40L on IL-21^+^ and IL-21^−^ NKT cells.

To observe expression of CD40L on surface of CD3^+^TCRvβ11^+^ NKT cells before and after culture with PPD plus anti-CD28, freshly isolated PFMCs were stained with anti-CD40L mAb before stimulation or together with anti-IL-21 mAb after stimulation. As shown in Figure 5A, without any stimulation, CD3^+^TCRvβ11^+^ NKT cells from PFMCs could express neither IL-21 nor CD40L. However, after stimulation with PPD plus anti-CD28, CD3^+^TCRvβ11^+^ NKT cells expressed both IL-21 and CD40L, and IL-21-expressing CD3^+^TCRvβ11^+^ NKT cells co-expressed CD40L. Statistical results in Figure 5B showed that the frequency of CD40L-expressing CD3^+^TCRvβ11^+^ NKT cells increased after stimulation, and Figure 5C illustrates that almost all of IL-21-producing CD3^+^TCRvβ11^+^ NKT cells expressed CD40L.

### PPD-specific IL-21-expressing CD3^+^TCRvβ11^+^ NKT cells in PFMCs expressed BCL-6 with higher frequency than non-IL-21-expressing CD3^+^TCRvβ11^+^ NKT cells

**Figure 6:**
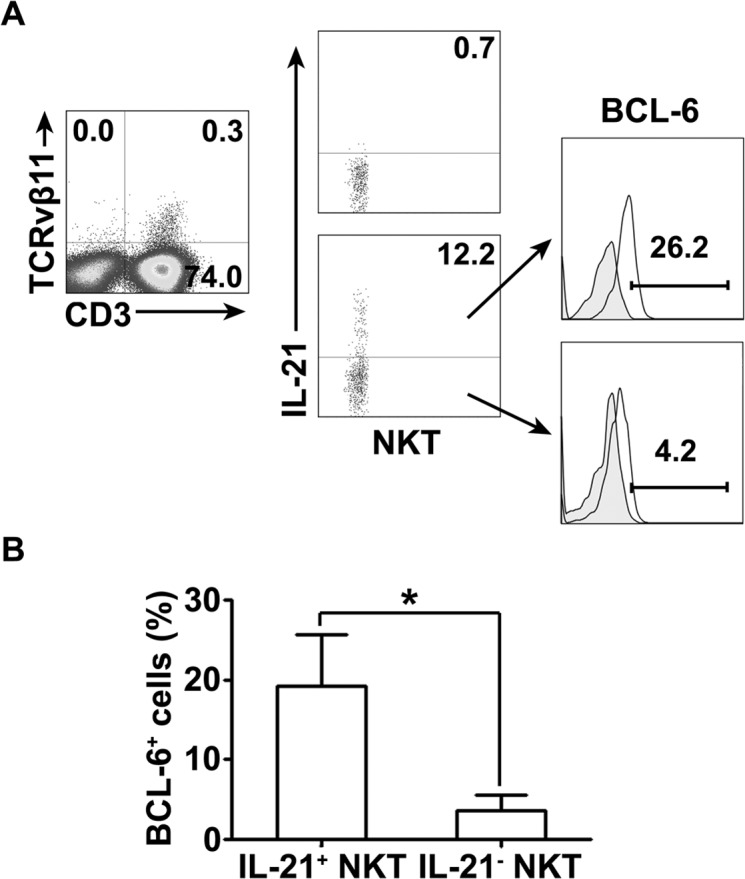
The expression of BCL-6 and IL-21 by NKT cells PFMCs were stimulated for 6 hrs with or without PPD plus anti-CD28 The cells were stained, gated and analyzed for the expression of BCL-6 and IL-21 on NKT cells. **A.** The expression of BCL-6 on IL-21^+^ and IL-21^−^ NKT cells was measured by FACS. Data are representative of three independent experiments. **B.** Statistical results of the expression of BCL-6 on IL-21^+^ and IL-21^−^ NKT cells. *, *P* < 0.05.

To detect whether IL-21-expressing NKT cells express BCL-6, freshly isolated PFMCs were cultured with PPD plus anti-CD28. CD3^+^TCRvβ11^+^ NKT cells were gated for expression of IL-21. IL-21- expressing NKT cells and non-IL-21- expressing NKT cells were gated for the analysis of expression of BCL-6. The results in Figure 6A showed that after stimulation with PPD plus anti-CD28, CD3^+^TCRvβ11^+^ NKT cells expressed IL-21, and IL-21-expressing NKT cells and non-IL-21- expressing NKT cells coexpressed certain levels of BCL-6. Statistical results in Figure 6B demonstrated that after stimulation nearly 20% of CD3^+^TCRvβ11^+^ NKT cells expressing IL-21 also expressed BCL-6, which was significantly greater than the frequency of BCL-6-expressing cells among non-IL-21- expressing NKT cells.

### Sorted CD3^+^TCRvβ11^+^ NKT cells from PFMCs produced IFN-γ and IL-21 and helped B cells to secrete IgG and IgA

**Figure 7:**
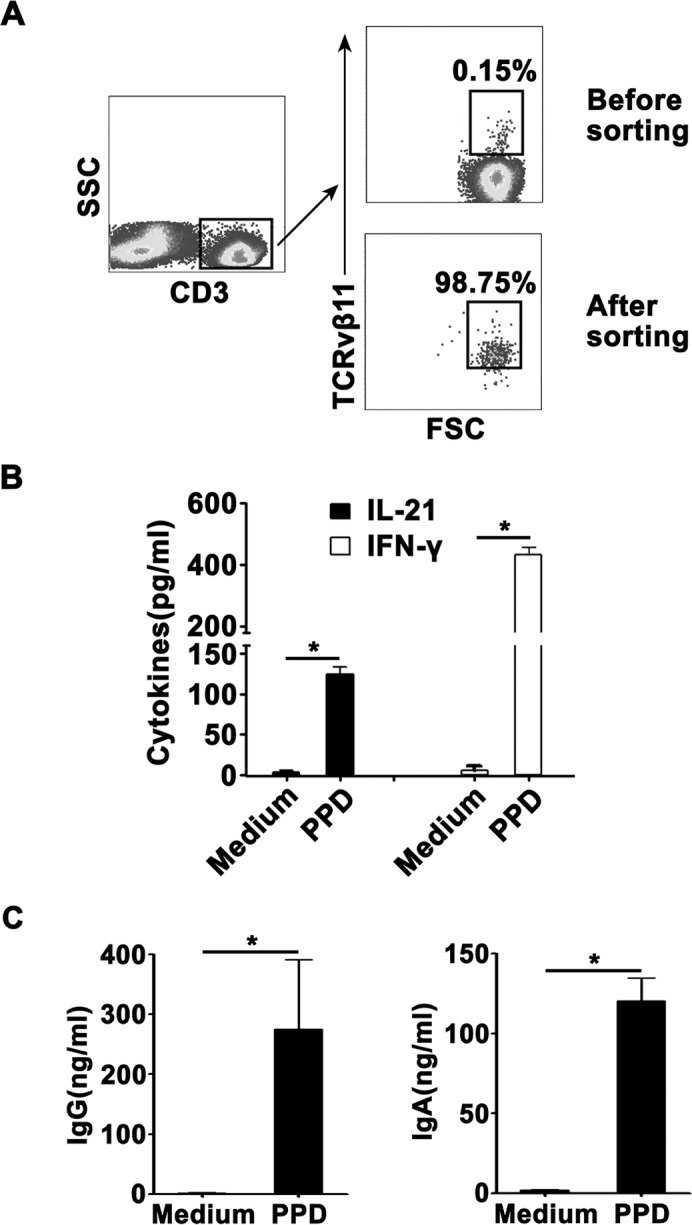
Sorted NKT cells expressed IL-21 and promoted the production of Igs by B cells NKT cells were sorted from PFMCs by FACS Sorted NKT cells were cultured with purified monocytes and stimulated for 10 days with PPD or Mtb-HAg. The production of IL-21, IFN-γ and IgG and IgA was measured by ELISA. **A.** NKT cells were sorted from PFMCs. The purity of sorted NKT cells is shown. **B.** Statistical results of the expression of IL-21 and IFN-γ by sorted NKT cells. **C.** Statistical results of the production of IgG and IgA by B cells cultured with sorted NKT cells. *, *P* < 0.05.

To further define the functions of CD3^+^TCRvβ11^+^ NKT cells, we isolated PFMCs and sorted purified CD3^+^TCRvβ11^+^ NKT cells by FACs. The results in Figure 7A showed that before the sorting, the frequency of CD3^+^TCRvβ11^+^ NKT cells was about 0.15%; but after the sorting, the purity of CD3^+^TCRvβ11^+^ NKT cells was nearly 99%. We co-cultured sorted CD3^+^TCRvβ11^+^ NKT cells and monocytes for 3 days with PPD plus anti-CD28 and then collected the supernatants to detect the production of IFN-γ and IL-21. Results in Figure 7B showed that without stimulation, CD3^+^TCRvβ11^+^ NKT cells did not secrete cytokines, and following stimulation with PPD plus anti-CD28, CD3^+^TCRvβ11^+^ NKT cells produced both IFN-γ and IL-21. Furthermore, we cultured sorted CD3^+^TCRvβ11^+^ NKT cells together with purified B cells to observe functions of CD3^+^TCRvβ11^+^ NKT cells. As shown in Figure 7C, purified B cells could secrete large amounts of IgG and IgA when cultured with CD3^+^TCRvβ11^+^ NKT cells. Based on the results, we speculate that at the local site of *M. tuberculosis* infection, CD3^+^TCRvβ11^+^ NKT cells secrete Th1 cytokines (IFN-γ, TNF-α and IL-2), Th17 cytokines (IL-17), and IL-21 and help B cells express Igs in order to protect against *M. tuberculosis* infection.

## DISCUSSION

Natural killer T (NKT) cells are a small population of thymus-derived T cells that are found in mice and humans and are restricted by non-classical MHC-I molecule CD1d. The term NKT cell initially referred to any T cell that expresses cell surface antigens associated with the NK cell lineage, but the classification of NKT cells is problematic because it does not define a T cell lineage with unique phenotypical or functional attributes. Even among CD1d-restricted T cells, distinct lineages that can express NK cell antigens include type 1 NKT cells (also known as invariant NKT (iNKT) cells), which express the invariant Vα24-Jα18 TCR α-chain, and type 2 NKT cells, which have a more diverse TCR repertoire. NKT cells express a semi-invariant TCR which confers a specificity for glycolipid antigens that are mainly conserved between mice and humans. It is known that after stimulation, NKT cells can produce large amounts of cytokines that can alter the strength and character of immune responses through crosstalk with dendritic cells, neutrophils, lymphocytes and myeloid-derived suppressor cells, and by shifting cytokine responses to (or from) T helper 1 (T_H_1), T_H_2 or T_H_17 cell type profiles. Expression of various kinds of cytokines exerts NKT cell immunoregulatory potential.

NKT cells are different from functionally differentiated conventional T cells in that they are autoreactive and produce both Th1 and Th2 cytokines, including IL-4, IL-10, and IFN-γ, upon stimulation with their ligands. Pediatric TB was associated with elevated plasma transforming growth factor β (TGF-β), IL-21, and IL-23 levels, which suggested that these responses might play a crucial role in the pathogenesis of tuberculosis. It was demonstrated that mycobacterium-specific Th17 cells formed a T cell subset that was distinct from cells producing the Th1 cytokines. Previous reports from our lab illustrated that ESAT-6-, CFP-10- or BCG-specific Th22 and Th17 cells were distinct from each other and from Th1 cells [30]. Our results showed that NKT cells from PBMCs and PFMCs produced IL-21, IFN-γ and IL-17, and that NKT cells from PFMCs produced IFN-γ, TNF-α, IL-2 and IL-17 in response to PPD and TB antigens (data not shown). Therefore, we draw the conclusion that NKT cells played a role in the immune responses against *M. tuberculosis* infection through production of those cytokines.

It was reported that IFN-γ-producing CD4^+^ and γδ T cells were effector memory cells with a phenotype of CD45RO^+^CD62L^−^CCR7^−^ [31, 32], which suggested that these cells could respond promptly to antigenic stimulation. Our results showed that in pleural fluids of TB patients, the majority of IL-21-producing NKT cells were also effector memory cells with a phenotype of CD45RO^+^CD62L^−^CCR7^−^. The NKT cells that could not produce IL-21 were partially central memory cells with a phenotype of CD45RO^+^CD62L^+^CCR7^+^. Central memory cells are thought to be long-lived populations, able to expand extensively for an effective secondary immune response. Taken together, our data suggest that IL-21-producing NKT cells in pleural fluids are effector memory cells and NKT cells that cannot express IL-21 are partially central memory cells with the potential to contribute to long-lasting protection against TB. Elucidating the mechanisms by which these NKT cell subsets might control *M. tuberculosis* infection is the subject of ongoing research.

It is known that Ag-cognate interactions occur between a particular subset of CD4^+^ Th cells, defined as T follicular helper (T_FH_) cells, and B cells and depend upon the engagement between CD40L (CD154) on T_FH_ cells and CD40 on B cells and upon the local cytokine environment created by the activated T_FH_ cells [33]. The expression of CXCR5 and production of IL-21 enable T_FH_ cells to migrate to B cell follicles, engage with Ag-activated B cells, and support their proliferation and differentiation into germinal centers (GCs). Our results demonstrated that nearly all of IL-21-producing NKT cells expressed CD40L and CXCR5, which is partially consistent with previous reports and may enable interactions between NKT cells and B cells.

We hypothesize that NKT cells play a role in bridging the innate and adaptive immune responses *via* their production of IL-21. Although IL-21 can enhance NK cell function, it also increases the death of NK cells [34, 35], increases inhibitory NK receptor expression on both NK [35] and NKT cells (data not shown), and down-regulates costimulatory molecule expression on dendritic cells (DC) [36, 37]. Conversely, because IL-21 enhances Ag specific Ab production [38, 39], CTL responses [34, 40] and the generation of central and effector memory T cells [41, 42]. Thus, NKT cell-derived IL-21 is an important factor underlying the well-established ability of NKT cells to enhance Ag-specific Ab production and CTL responses [43-45]. Our findings demonstrated NKT cells from the pleural fluid of TB patients produced IL-21 after stimulation with PPD and IL-21 helped B cells to secrete IgG and IgA. These results suggest that, in addition to producing Th1 and Th2 cytokines, NKT cells also induce B cells to secrete Igs through an IL-21-dependent mechanism and can participate in local immune responses against *M. tuberculosis* infection.

In summary, we found that *M. tuberculosis*-specific antigens induced production of IL-21 in addition to IFN-γ, TNF-α, IL-2 and IL-17 by NKT cells in pleural fluids from TB patients. IL-21-expressing NKT cells expressed phenotypes of effector memory cells and co-expressed CXCR5 and CD40L. Furthermore, IL-21 produced by NKT cells promoted B cells to secrete IgG and IgA. Our data deepen information on local immune responses against *M. tuberculosis* infection and provide a further rationale for the evaluation of NKT cells in host defense against tuberculosis.

## MATERIALS AND METHODS

### Study participants

Twenty patients with tuberculous pleurisy (10 females and 10 males, 23-71 years old) were recruited from the Chest Hospital of Guangzhou, China. The diagnosis of pleural effusion from TB etiology was based on the following criteria: (i) *M. tuberculosis* on a pleural fluid smear (by Ziehl-Neelsen method); (ii) pleural fluid or pleural biopsy specimens growing *M. tuberculosis* on Lowenstein-Jensen medium; (iii) histological evidence of caseating granuloma on biopsy specimens of pleural tissue with positive staining for *M. tuberculosis*. Patients with HIV, HBV, HCV or a history of autoimmune diseases were excluded from the study. Twenty healthy volunteers (10 females and 10 males, 23-60 years old) were recruited from Sun Yat-sen University. Written informed consent was obtained from all patients and healthy donors. Ethics approval for the present study was obtained from the ethics committee of the Zhongshan School of Medicine, Sun Yat-sen University (Guangzhou, China) and the Chest Hospital of Guangzhou (Guangzhou, China).

### Antigens and antibodies

Purified protein derivative (PPD) was kindly provided by Aeras Global TB Vaccine Foundation (USA). The purity of PPD was > 90%. *Mycobacterium tuberculosis* heat resistant antigen of low molecular weight peptide (Mtb-HAg) was kindly provided by professor Baiqing Li from Bengbu Medical College (Bengbu, China). Purified anti-CD28 mAb (CD28.2) was purchased from BD Biosciences Pharmingen. The following panels of anti-human mAbs were used for analyses of cell surface markers and intracellular cytokines: PE-cy7-conjugated anti-CD3 (SK7), phycoerythrin (PE)-conjugated anti-CD3 (UCHT1), allophycocyanin (APC)-conjugated anti-CD3 (SP34-2), PE-CF594-conjugated anti-CD3 (UCHT1), PE-conjugated anti-CD4 (RPA-T4), APC-conjugated anti-CD4 (RPA-T4), APC-Cy7-conjugated anti-CD4 (RPA-T4), PE-conjugated anti-CD8 (RPA-T8), APC-conjugated anti-CD8 (RPA-T8), PE-conjugated anti-CD45RO (UCHL1), Alexa Fluor700-conjugated anti-CD45RO (UCHL1), APC-conjugated anti-CD62L (DREG-56), PE-cy7-conjugated anti-CCR7 (3D12), Alexa Fluor647-conjugated anti-CXCR5 (RF8B2), PE-conjugated anti-CD25 (M-A251), PE-Cy7-conjugated anti-CD69 (FN50), PE-conjugated anti-CD40L (TRAP1), APC-conjugated anti-CD40L (TRAP1), APC-conjugated anti-IFN-γ (B27), PE-cy7-conjugated anti-IFN-γ (4S.B3), PE-conjugated anti-TNF-α (MAb11), PE-cy7-conjugated anti-TNF-α (MAb11), PE-conjugated anti- IL-2 (MQ1-17H12), APC-conjugated anti- IL-2 (MQ1-17H12), PerCP-cy5.5-conjugated anti-IL-2 (MQ1-17H12), PE-conjugated anti-IL-21 (3A3-N2.1) (BD Biosciences, San Jose, CA, USA), Alexa Fluor647-conjugated anti-IL-21 (3A3-N2) (eBioscience, San Diego, CA, USA) and fluourescein isothiocyanate (FITC)-conjugated anti-TCRvβ11 (C21) (Beckman Coulter, Brea, CA, USA).

### Preparation of PFMCs and PBMCs

PFMCs were isolated by lysing erythrocytes with an ammonium chloride solution and resuspending the pellet to a final concentration of 2×10^6^ /mL in complete RPMI 1640 medium (Gibco, Grand Island, NY, USA) supplemented with 10% heat-inactivated fetal calf serum (Sijiqing, Hangzhou, China), 100 U/mL penicillin, 100 μg/mL streptomycin, 2 mM L-glutamine and 50 mM 2-mercaptoethanol. Peripheral blood mononuclear cells (PBMCs) were isolated from heparinized venous blood by Ficoll-Hypaque (Tianjin Haoyang Biological Manufacture, Tianjin, China) density gradient centrifugation within 24 hours of blood drawing. The cells were collected and washed twice in Hank's balanced salt solution. Viability of the cells was tested using trypan blue dye exclusion. The cells were finally resuspended at a final concentration of 2×10^6^ /mL in complete RPMI 1640 medium.

### Isolation of monocytes and T cells

CD14^+^ cells were positively purified from freshly isolated PFMCs by use of anti-CD14 microbeads (Miltenyi Biotec, Bergisch Gladbach, Germany) according to the manufacturer's protocol. Briefly, after washing twice in magnetic activated cell sorter (MACS) buffer (phosphate-buffered saline[PBS] supplemented with 2 mM EDTA and 0.5% bovine serum albumin [BSA]), PFMCs were resuspended in the buffer, mixed well with anti-CD14 microbeads and incubated at 4°C for 15 min. The cells were washed and magnetically separated on a MACS magnet fitted with a MACS LS column. The purity of monocytes was > 97%, as assessed by flow cytometry. Unlabeled cells were collected and used for the purification of T cells. In brief, the cells were washed twice with buffer and resuspended in buffer, mixed well with anti-CD3 microbeads (Miltenyi Biotec, Bergisch Gladbach, Germany), and incubated at 4°C for 15 min. After washing, the cells were resuspended in buffer and magnetically separated with an LS column. The purity of T cells was > 97%, as detected by flow cytometry.

### Cell sorting

Purified T cells from PFMCs were stained with FITC-labelled anti-TCRvβ11 mAb, and non-T cells were stained with PE-labelled anti-CD19 mAb for 30 min at 4°C in the dark and washed twice with the buffer as described above. The cells were suspended in the buffer and sorted using a FACS Aria II flow cytometer (Becton Dickinson, San Jose, CA) for TCRvβ11^+^ cells and B cells from PFMCs. The purity of TCRvβ11^+^ cells was > 98%, and of the B cells was > 99%.

### Flow cytometric analysis of cell-surface markers and intracellular cytokine staining

The cells were washed twice with PBS buffer containing 0.1% BSA and 0.05% sodium azide. For surface staining, cells were incubated with the respective mAbs at 4°C in the dark for 30 min. Cells were washed twice and fixed in 0.5% paraformaldehyde before acquisition. For the detection of intracellular cytokines, cells were incubated with PPD or Mtb-HAg plus anti-CD28 mAb for 6-8 hrs in the presence of brefeldin A (10 μg/mL; Sigma-Aldrich, St Louis, MO). After stimulation, cells were washed twice with PBS and fixed in 4% paraformaldehyde, followed by permeabilization, and stained for the intracellular cytokines and molecules in PBS buffer containing 0.1% saponin. Lymphocytes were gated on forward- and side-scatter profiles. Flow cytometry was performed using a BD FACS Calibur (Becton Dickinson, San Jose, CA) and analyzed using FlowJo software (TreeStar, San Carlos, CA).

### ELISA assay

Sorted CD3^+^TCRvβ11^+^ NKT cells with or without purified monocytes were resuspended in complete RPMI 1640 medium and cultured in the presence or absence of anti-CD3 plus anti-CD28 mAbs or phorbol myristate acetate (PMA) plus Ionomycin for 48-72 hrs. The supernatants were harvested and assayed for the production of IFN-γ (ELISA; detection limit, 4.7 pg/mL) (BD Pharmingen, San Diego, CA) and IL-21 (ELISA; detection limit, 46 pg/mL) by enzyme-linked immunosorbent assay (ELISA) according to the manufacturer's protocol. Sorted CD3^+^TCRvβ11^+^ NKT cells with or without sorted B cells were resuspended in complete RPMI 1640 medium and cultured in the presence or absence of PPD or Mtb-HAg for 10 days. The supernatants were harvested and assayed for the production of IgG (detection limit, 7.8 ng/mL) and IgA (detection limit, 7.8 ng/mL) by ELISA according to the manufacturer's protocol (eBioscience, San Diego, CA, USA).

### Statistical analysis

All statistical tests were performed with GraphPad Prism 5 (GraphPad Software Inc, San Diego, CA, USA). Differences between groups were assessed by the Kruskal-Wallis test with Dunn's multiple comparison test. A value of *P* < 0.05 was considered significant.
